# Functional and Morphological Characteristics of the Retina of Patients with Drusen-like Deposits and Systemic Lupus Erythematosus Treated with Hydroxychloroquine: A Retrospective Study

**DOI:** 10.3390/biomedicines11061629

**Published:** 2023-06-03

**Authors:** Alice M. Kitay, James V. M. Hanson, Nasiq Hasan, Matthew Driban, Jay Chhablani, Daniel Barthelmes, Christina Gerth-Kahlert, Mayss Al-Sheikh

**Affiliations:** 1 Department of Ophthalmology, University Hospital Zurich, University of Zurich, Frauenklinikstrasse 24, 8091 Zurich, Switzerland; alice.kitay@usz.ch (A.M.K.); james.hanson@usz.ch (J.V.M.H.); daniel.barthelmes@usz.ch (D.B.); christina.gerth-kahlert@usz.ch (C.G.-K.); 2 UPMC Eye Center, University of Pittsburgh, Pittsburgh, PA 15213, USA; nasiq.imtiaz@gmail.com (N.H.); driban.matthew@medstudent.pitt.edu (M.D.); jay.chhablani@gmail.com (J.C.); 3 Save Sight Institute, The University of Sydney, Sydney, NSW 2006, Australia

**Keywords:** drusen-like deposits, systemic lupus erythematosus, hydroxychloroquine, multifocal electroretinography, optical coherence tomography

## Abstract

Purpose: To evaluate the impact of drusen-like deposits (DLD) on retinal layer integrity and retinal function by optical coherence tomography (OCT) and multifocal electroretinography (mfERG) in patients with systemic lupus erythematosus (SLE). Methods: We identified 66 eyes of 33 SLE patients treated with hydroxychloroquine (HCQ) that were categorized into two groups according to whether DLDs were present (34 eyes, Group One) or absent (32 eyes, Group Two). The groups were matched for age, sex, HCQ treatment duration, daily, and cumulative dosage. OCT (retinal layer thicknesses, central retinal thickness, CRT) and mfERG concentric ring analysis were analyzed and compared. Results: CRT was significantly thicker in Group One compared to Group Two (273.21 ± 3.96 vs. 254.5 ± 7.62) (*p* = 0.023). Group One also demonstrated an overall thicker retinal pigment epithelium compared to Group Two; however, the other outer retinal layers, outer nuclear layer, and photoreceptor layer were found to be significantly thinner in Group One compared to Group Two. We found no differences in mfERG parameters between the two groups. Conclusions: DLDs in SLE patients lead to abnormal central retinal layer thickness, which has no measurable impact on cone-mediated retinal function assessed by mfERG.

## 1. Introduction

Systemic lupus erythematosus (SLE) is a chronic disorder of unknown underlying etiology leading to the production of autoantibodies against connective tissue that affects multiple organs of the human body [1]. The clinical course is dynamic, characterized by relapses and periods of remission. The American Rheumatologists Association (ARA) determined eleven criteria, of which a minimum of four of the following must be present to fulfill the diagnosis of SLE: photosensitivity, malar or discoid rash, non-erosive arthritis, oral ulcers, renal dysfunction, serositis, presence of antinuclear antibodies, and neurological (i.e., seizures), hematological (i.e., anemia), or immunological (i.e., anti-DNA antibody) signs [1].

**Figure 1:**
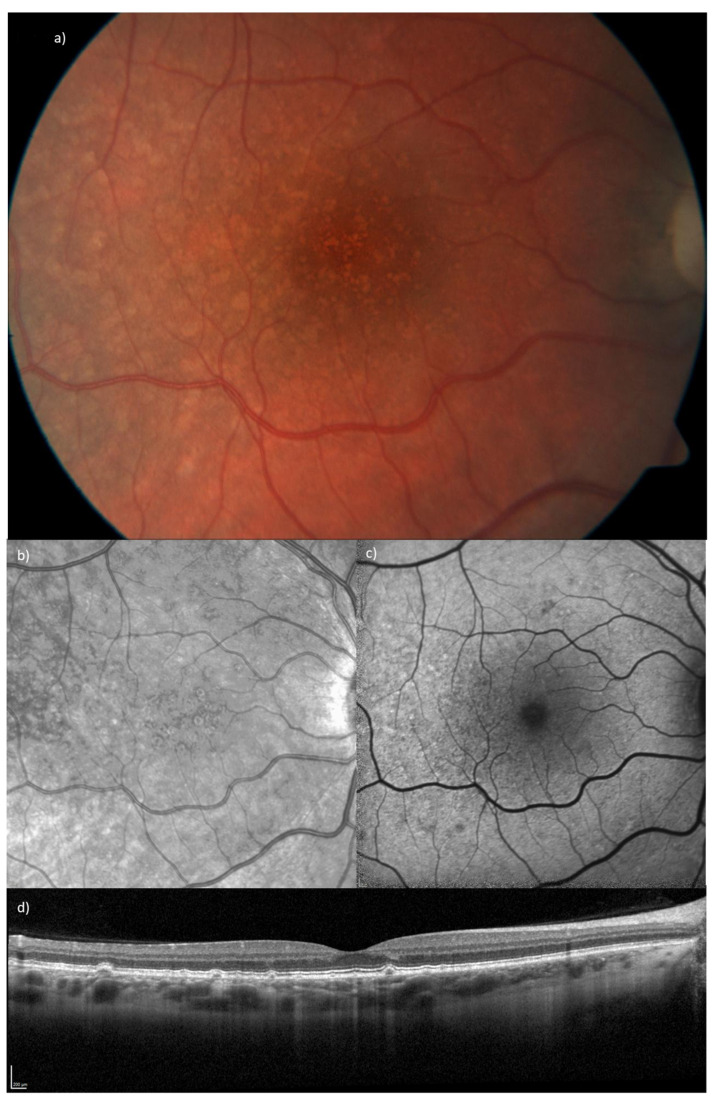
Right eye fundus images of a 26-year old female SLE patient with a long-term HCQ treatment (cumulative dose of 973g). (**a**) ZEISS fundus photography of the posterior pole demonstrates yellowish drusenoid alterations of the RPE. (**b**) Near infrared (NIR) image and (**c**) Fundus autofluorescence of the posterior pole. (**d**) SD-OCT scan through the central fovea with dome-shaped subretinal drusen-like deposits with alterations of the outer retina and RPE.

SLE can manifest with a broad range of ocular problems. Lupus retinopathy is a vision-threatening complication following vascular occlusion, choroidal vasculitis, and serous retinal detachment. It is associated with active renal and/or CNS involvement and is therefore seen in advanced disease stages [2]. Newer retinal associations are drusen-like deposits (DLD), funduscopically seen as yellowish drusenoid subretinal alterations, located between the retinal pigment epithelium (RPE) and Bruch’s membrane [3]. Early studies based on fluorescein and indocyanine green angiography described DLDs as rare findings seen in patients with glomerulonephritis underlying SLE, suggesting an anatomical similarity of the choriocapillaris-Bruch-RPE complex and the glomerulus [4]. However, the work of Invernizzi et al. documented with an extensive OCT analysis the presence of small DLDs in young SLE patients without renal involvement [3]. The group postulated an association between DLDs and complement pathways dysregulation in the capillary system of the eye. In addition, their findings are attributable to the technical advantages of modern imaging techniques [3]. Histologically, DLDs have been demonstrated to react with antibodies against C5, TIMP3, vitronectin, and amyloid P component, which are the markers of age-related macular degeneration (AMD)-associated drusen, thus outlining a similarity between DLD and the typical drusen seen in patients with AMD. Interestingly, DLDs can be distinguished from drusen by fundus autofluorescence (FAF) imaging, as DLDs do not show hyperautofluoresence, as demonstrated in Figure 1c [5]. Until now, the prevalence and clinical relevance of DLDs remain poorly understood.

SLE patients with isolated DLDs do not have any noticeable symptoms, and the retinal changes are often diagnosed by chance during ophthalmological screening initiated due to hydroxychloroquine (HCQ) treatment. The antimalarial drug HCQ is considered the standard therapy for patients with SLE due to its efficacy and good patient tolerance [6]. In addition, it can modify several immunological pathways and is therefore used as an immunosuppressant for many rheumatological and autoimmune diseases. However, possible side effects of HCQ include irreversible damage to the retina, specifically the photoreceptor layer and the retinal pigment epithelium [7]. Hence, the American Academy of Ophthalmology recommends ophthalmological assessment, including morphological and functional testing, i.e., spectral-domain optical coherence tomography (SD-OCT) and multifocal electroretinogram (mfERG) at baseline and for periodical follow-up visits [8]. In addition, Drusen, mainly in AMD, has been described to be associated with general retinal dysfunction detectable by mfERG [9]; however, the influence of DLDs on mfERG responses and, therefore, the possible influence on this screening modality has not yet been assessed.

The objective of this study was to evaluate functional changes in patients with DLD compared with SLE patients without DLD using mfERG, in addition to morphological changes using SD-OCT. Accordingly, we hypothesized that DLDs would influence mfERG responses and increase central retinal thickness.

## 2. Methods

### 2.1. Patient Inclusion

This retrospective and comparative cohort study was performed at the Department of Ophthalmology, Zurich University Hospital, during the period January 2012–December 2020. This study adhered to the tenets of the Declaration of Helsinki, and patients provided informed written consent. Furthermore, the study protocol was approved by the Institutional review board of Swiss Ethics/BASEC (No. 2019-00972).

Inclusion criteria were the following: male or female subjects aged ≥18 years with the diagnosis of SLE. Included patients came for regular examinations in the HCQ screening clinic; thus, all of the included patients were on HCQ therapy at the time of examination. Patients were excluded from the analysis if other retinal conditions (including HCQ retinopathy) or any ocular conditions requiring (potentially repetitively) surgical intervention, such as corneal diseases or glaucoma, were present. Patient data were also excluded from analysis if factors affecting visual acuity measurement were present, e.g., cataract, previous anterior segment trauma.

### 2.2. Data Collection

All patients underwent a comprehensive ophthalmological examination, including Snellen visual acuity assessment, air or Goldmann tonometry, slit lamp examination, and fundoscopy on dilated pupils. In addition, SD-OCT (HEYEX 2^®^, Heidelberg Engineering, Heidelberg, Germany) and mfERG (Espion^®^, Diagnosys LLC, Lowell, MA, USA) were performed on each patient. Other recommended tests, such as static perimetry, FAF, and fundus photography, were performed but not included in the analysis. Clinical information obtained included a detailed medical history of the underlying disease, patient’s weight, and history of HCQ treatment (daily dosage of HCQ, duration of treatment, cumulative dosage).

**Figure 2:**
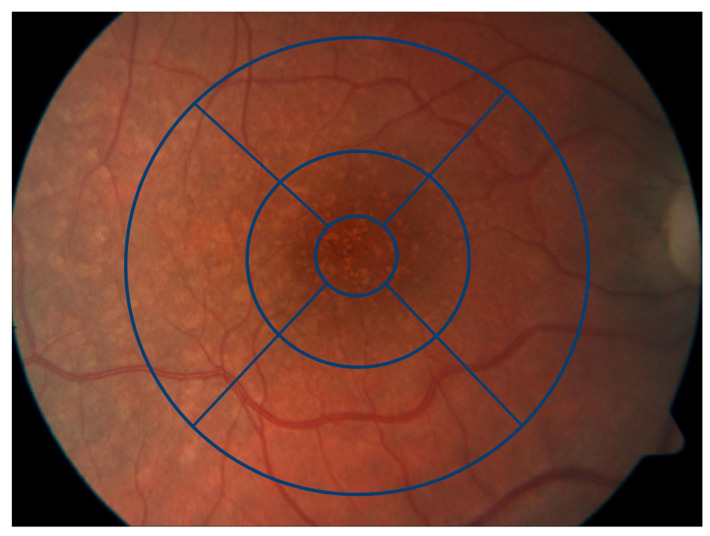
Distribution of the drusen-like deposits was defined by the ETDRS grid in a central area, including the fovea with a diameter of 1 mm, an inner ring with a diameter of 3 mm, and an outer ring with a diameter of 6 mm.

SD-OCT enabled us to thoroughly analyze and assess differences in the retinal layers of the macula and determine its thickness using a minimum of 31 horizontal B-scans (248 µm interscan distance) with a minimum of 10 automatic real-time tracking (ART) frames averaged. OCT and other imaging findings in an SLE patient with DLD are illustrated in Figure 1 Analysis of the thickness of individual and aggregated retinal layers was conducted with the built-in software HEYEX 2 using the automatic segmentation function. This permits the generation of thickness maps, which we used for an outer retinal layer study, looking specifically at the RPE, ONL, and photoreceptor layer over the standardized Early Treatment Diabetic Retinopathy Study (ETDRS) grid. The ETDRS grid represents the accepted standard for the classification and measurement of retinal thickness [10]. Thicknesses of the RPE, ONL, and photoreceptor layer of the nine different areas of the macula defined by the grid (see Figure 2) were compared between groups. We also calculated the sum of all four areas in the 3 mm and 6 mm regions and compared them between the groups (see Table 1 and Table 2). All OCT scans and the automatically segmented retinal layer boundaries were checked for possible segmentation artifacts and algorithm failures, and manually corrected when necessary [11].

**Figure 3:**
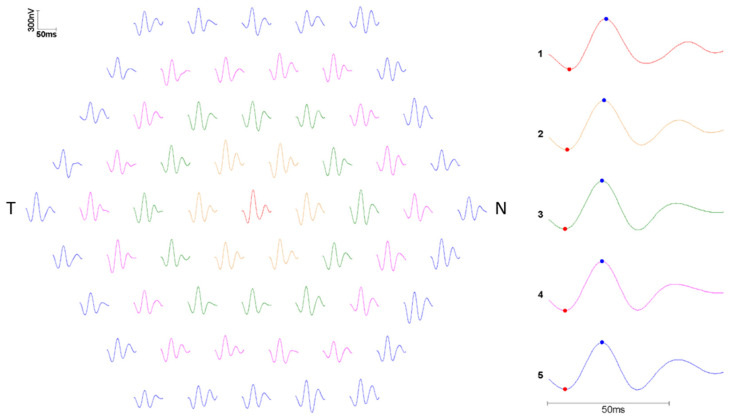
Example of the multifocal electroretinography (mfERG) concentric ring analysis of a right eye of a patient with drusen-like deposits (DLD) (N = nasal, T = temporal). Colored traces demonstrating the mfERG responses grouped by concentric rings R1 (red, central)—R5 (blue, peripheral) of a DLD patient.

MfERG was recorded using single-use DTL (Dawson, Trick and Litzkow)-type recording electrodes (Diagnosys LLC, Lowell, MA, USA) and gold-plated skin electrodes according to contemporary published standards of the International Society for Clinical Electrophysiology of Vision [12], as described elsewhere [13]. Medical mydriasis was accomplished using topical 0.5% tropicamide and 5% phenylephrine. Prior to applying the skin electrodes (reference electrodes at the ipsilateral outer canthi; ground electrode at the center of the forehead), patients’ skin was cleaned with alcohol-based hand disinfectant and scrubbed using an abrasive paste, in order to minimize electrical impedance during recording. In addition, to prevent potential patient discomfort, 0.4% oxybuprocaine was instilled prior to installing and positioning the DTL electrodes. Recordings were made using an achromatic 61 hexagon stimulus array covering approximately 50° of the central visual field in normal room illumination. Hexagons had a luminance of either 0.0 cd/m^2^ (‘off’) or 400 cd/m^2^ (‘on’), with the off/on string of each hexagon determined according to a 14-bit M-sequence. The base period for stimulus presentation was 13.3 ms (equivalent to 75 Hz), and the recordings were bandpass filtered (10–100 Hz) in order to remove extraneous electrical noise. Each recording session lasted 30 s, with a minimum of eight sessions required to complete the mfERG recording. MfERG P1 amplitudes were analyzed using the concentric ring method, demonstrated in Figure 3 [14]. Additionally, the amplitude ratios of rings 1, 2, 3, and 4 relative to ring 5 were calculated and included in the analysis [15]. Therefore, a total of nine mfERG parameters were analyzed: five amplitudes and four ring ratios.

### 2.3. Statistical Analysis

Stata 16 (StataCorp (2019), College Station, TX, USA) was used to analyze data. Summary statistics included mean and standard deviation for continuous variables and frequencies with percentages for categorical variables. Clinical, SD-OCT, and mfERG results were analyzed as proportions, mean, and standard deviations. The median and interquartile range were determined in case of skewed data. The cohort was subdivided into two groups: SLE patients with DLD and SLE patients without DLD. We then performed the two-sample t-test, chi-squared test, and analysis of covariance (ANCOVA) [16,17,18] to exemplify significant differences between both groups. Multivariate logistic regression, adjusted for age, sex, and cumulative HCQ dose, was performed to test for associations between the presence of DLD and cone-mediated retinal function. All results with a *p*-value < 0.05 were considered to be significant.

## 3. Results

### 3.1. Demographics

In this study, we included 66 eyes of 33 patients with SLE and HCQ therapy. A total of 34 eyes of 17 patients were diagnosed with DLDs and considered subjects (Group One), whereas 32 eyes of 16 patients who did not have any fundus abnormalities were taken as controls (Group Two). The demographic data and clinical characteristics of patients are shown in Table 1. Patients in the two groups were equally matched for age, sex, daily and cumulative dose at examination, and treatment duration. The mean age was 41.95 ± 12.15 years in Group One and 40.10 ± 12.63 years in Group Two (*p* = 0.69). The majority of the patients included in this study were female and Caucasian. The mean daily dose of HCQ was 250.00 ± 89.44 mg in group one and 287.50 ± 102.47 mg in Group Two (*p* = 0.28). The mean HCQ treatment duration was 8.20 ± 5.44 years in Group One and 6.10 ± 4.89 years in Group Two (*p* = 0.24).

### 3.2. SD-OCT Analysis

Detailed SD-OCT evaluation revealed significant differences in different layers of the outer retina, and at different retinal areas, the OCT scan was obtained. In the analysis of covariance (ANCOVA), including age, sex, and cumulative HCQ dose as covariates, the central foveal thickness was determined to be significantly thicker in Group One (273.21 ± 3.96 vs. 254.5 ± 7.62, *p* = 0.023, R2 = 0.1102). The other areas within the 3 mm and 6 mm rings on the analysis implemented on the OCT scan were similar between the two groups.

The RPE was found to be significantly thicker in nearly all areas of Group One than in Group Two (Table 2), whereas the photoreceptor layer was found to be significantly thinner in Group One compared to Group Two (Table 3).

### 3.3. Mf-ERG Analysis

The concentric ring analysis of the mfERG studies did not show significant differences in amplitude in the five different rings in both groups. The ring ratios were also comparable between Group One and Group Two. In addition, multivariate logistic regression adjusted for age, sex, and cumulative HCQ dose identified no significant associations between the presence of DLD and increased or decreased mfERG amplitude in any ring (*p* > 0.05 for each ring). Thus, we did not observe any significant differences in mfERG parameters between the two groups (Table 4).

## 4. Conclusions/Discussion

The objective of this study was to evaluate the functional and morphological characteristics of SLE patients presenting with DLDs. We used the well-established diagnostic tools OCT and mfERG to evaluate differences in the outer retina and photoreceptor function. In this study, we investigated morphological and functional features in the retina depending on the presence of DLD in patients with SLE. For this purpose, OCT-derived retinal layer thicknesses and macular function (as measured with mfERG) were compared in SLE patients with and without documented DLD.

Our key result in the OCT analysis is the significant increase of foveal outer retina thickness in patients with SLE and DLDs compared to patients with SLE without DLDs. Conversely, the photoreceptor cell layer displayed significant thinning in patients with DLD, which we attributed most likely to degeneration following inflammatory episode(s) with vasculitis and reduced blood flow.

Our results indicate an increase in central retinal thickness in SLE patients with DLDs. These results agree with those recorded by Invernizzi et al., who analyzed SD-OCT findings in SLE patients and detected a significant central thickness increase in patients with SLE compared to a healthy control cohort [3]. They also found a thicker choroid in patients with DLDs. The authors concluded that these findings were highly correlated with a systemic inflammatory state in SLE, and suggested the presence of DLDs as an indicator for inflammatory conditions.

However, our findings stand in contrast to previously published data, which highlighted a thinning of the central retinal thickness in patients with SLE compared to healthy subjects [16,17,18]. Liu et al. explained that the retinal thickness decreases as a consequence of vasculitis with subsequent atrophy of the inner retinal layers, including the RNFL and ganglion cell layer [18]. Work by Dias-Santos et al. suggested retinal neurodegeneration in the course of SLE disease progression, despite recording a concurrent photoreceptor layer thickness increase; the authors reconciled these findings by proposing neuronal remodeling as an explanation for the thickness increase [16]. The work by Jones et al. discussed these circumstances in more detail. The authors described the development of retinal neurodegeneration in patients with retinitis pigmentosa and described an unstructured glial reaction with new synaptic connections after initial retinal damage accompanied by apoptosis [19]. This remodeling is seen as a part of the degenerative process and may affect most of the retinal layers. Interestingly, a histopathological examination of a patient with SLE showed significant macrophage infiltration in the retina [19]. These molecular findings may explain the increased retinal layer thickness, as shown in our study.

Congruent with the findings by Dias-Santos et al., we found a significantly thinner photoreceptor cell layer in SLE patients compared to subjects without such lesions [16]. We suggest that these changes may be explained by the high metabolic rate and energy demand of this retinal area. The photoreceptors are dependent on the neighboring choriocapillaris for their metabolic requirements [16,20,21]. Therefore, SLE can adversely affect the choroidal structures and blood flow, leading to ischemia and atrophy in the retinal layers dependent on the choroid for oxygen and nutrients [16,22]. In this context, our results suggest that the existence of DLDs may be a sign of an advanced autoimmune disease state, as evidenced by the neurodegeneration of the outer retinal layers seen in optical coherence tomography scans.

DLD are new observational findings with as-yet unknown clinical relevance [3]. In contrast, the pathogenesis of AMD-related drusen is well understood. AMD-related drusen are defined as lipoprotein accumulation in the macular Bruch’s membrane that increase in size as the disease progresses, and their development is influenced by genetic and environmental factors [23]. They lead to a decrease in membrane conductivity and further oxygen stress, and local inflammation [24]. The distribution of AMD-derived drusen is ubiquitous on the whole posterior pole and beyond. The fovea is frequently not spared [25].

It is currently assumed that DLDs are accumulations of immunocomplexes and complement factors [3]. This assumption is underlined by the age at which patients are observed with DLDs, as they appear in younger patients with autoimmune diseases that involve the complement system [26]. Regarding the distribution, DLDs typically show a perifoveal arrangement with foveal sparing [3]. The distribution pattern was reported in diseases other than SLE, primarily in diseases in which immunocomplex accumulation and the activation of the complement system play a pivotal role. The incidence of DLD in renal diseases gives rise to a pathophysiological and anatomical congruence between the glomerular system and Bruch´s membrane-RPE complex. Furthermore, lupus nephritis patients with DLD manifestation demonstrated an advanced and active disease course, indicating DLD to be a prognostic factor regarding severity and progression in SLE patients [3,16]. Lastly, the detection of DLD could play an important role in diagnosing silent lupus nephritis, which can be detected with renal biopsies; however, conventional urinalysis and laboratory blood results show normal results [27]. The implementation of OCT as a diagnostical screening tool is, therefore, a cheap and non-invasive way with easy performance to identify SLE patients at risk for renal and visual affection.

The mfERG analysis did not show a significant difference in the amplitudes and signal transmission between both groups, despite our morphological finding of thinning of the photoreceptor (in which the mfERG originates). Thus, the presence of DLDs had no measurable impact on the mfERG recorded with a 61-hexagon stimulus array and analyzed using the standard concentric ring method. However, it is possible that recording the MF-ERG with a denser stimulus array would have enabled the detection of subtle differences between groups due to the greater spatial resolution; conversely, the reduced signal/noise ratio when using a denser stimulus array may have negated the theoretically beneficial effects of this increased spatial resolution [28]. Similarly, whilst comparing the amplitudes of individual traces across the two groups may have helped identify circumscribed areas of retinal dysfunction, the large data sets (61 traces per eye) rendered this impractical.

Earlier work by Gerth et al. demonstrated the impact of soft and large AMD drusen on mfERG. The authors found significantly abnormal responses in and around areas of drusen and described a morphological-functional relationship [9,29]. They proposed a dysfunction in the cone-driven pathways and the interference of the drusen on bipolar and photoreceptor cells due to an altered and delayed transmission [9]. The same group also demonstrated a progressive deterioration in the cone-driven mfERG response despite stable visual acuity and drusen morphology [29]. Of note, these authors used a stimulus array consisting of 103 hexagons (offering greater spatial resolution than the 61 hexagon array employed in our study), and contact lens electrodes (which provide a better signal-to-noise ratio compared to DTL electrodes). They analyzed responses at the level of the individual traces. In general, early changes in mfERG due to AMD consist of a delayed implicit time of the responses [30]. In advanced cases, mfERG amplitudes are typically reduced [30]. Furthermore, the response deterioration correlates with disease progression [30].

Since mfERG is an important diagnostic screening tool for clinicians to diagnose and/or exclude HCQ retinopathy, it is of high interest to know that DLDs in patients with SLE (who are very often on HCQ treatment) may not have an impact or falsifying effect on this screening modality [8].

Our study has limitations. The number of subjects included in this study is limited, and it followed a retrospective approach. In addition, retinal layer images and mfERGs were not compared to a separate healthy cohort (patients with no SLE and no HCQ treatment). In the future, a long-term follow-up study would be helpful to reevaluate possible changes and/or progression in patients with DLD and the impact of the morphological changes on retinal layer thickness and mfERG responses. We want to address these remarks in a future longitudinal study for definitive answers regarding DLD properties and progression, as well as their effect on visual outcomes.

In conclusion, this study may have implications for patients with SLE in terms of disease activity. The presence of DLD can be considered a disease activity indicator, as SLE patients with these retinal lesions demonstrate a more active disease, whereas SLE patients without DLD demonstrate a less active disease state. Active inflammation is expressed by increased outer retina thickness, reflecting vasculitis and immunocomplex deposition.

The thinning of the photoreceptor and ONL layer are consistent with secondary neurodegeneration, and reduced blood flow may be an indicator of disease progression. In summary, DLDs have a structural impact on retinal layer thickness, which appears to have no measurable impact on the functional transmission of the photoreceptor cells.

## Figures and Tables

**Table 1:** Demographic data.

Parameter	Group One	Group Two	*p* Value
Mean age in years ± SD	41.95 ± 12.15	40.10 ± 12.63	0.69
Sex	M = 4 F = 13	M = 5 F = 11	0.36
Ethnicity	C = 13 A = 4	C = 11 A = 5	0.36
Renal involvement	10 (58.8%)	3 (18.9%)	0.06
Daily dose (milligrams)	250.00 ± 89.44	287.50 ± 102.47	0.28
Cumulative dose (grams)	608.60 ± 361.48	646.16 ± 491.62	0.81
Treatment duration (years)	8.20 ± 5.44	6.10 ± 4.89	0.24

SD, standard deviation; M, male; F, female; C, Caucasian; A, Asian.

**Table 2:** Comparison of RPE thickness (in µm) in different ETDRS grid areas (in mm) in both groups.

Area	Group One	Group Two	*p* Value
Central 1	23.19 ± 21.38	18.59 ± 5.62	0.24
Nasal 3	20.38 ± 12.59	20.50 ± 21.68	0.98
Nasal 6	15.50 ± 4.66	13.36 ± 2.77	0.03
Superior 3	18.94 ± 5.99	16.25 ± 1.81	0.02
Superior 6	15.06 ± 2.67	13.78 ± 1.36	0.02
Temporal 3	19.09 ± 7.75	15.53 ± 1.50	0.01
Temporal 6	15.34 ± 2.66	13.41 ± 1.52	0.001
Inferior 3	18.91 ± 8.23	15.66 ± 1.41	0.03
Inferior 6	14.38 ± 2.74	13.53 ± 1.32	0.12
Sum of all areas within 3	77.81 ± 33.21	62.80 ± 11.03	0.02
Sum of all areas within 6	60.52 ± 11.99	53.07 ± 8.81	0.01

**Table 3:** Comparison of photoreceptor layer thickness (in µm) in different ETDRS grid areas (in mm) in both groups.

Area	Group One	Group Two	*p* Value
Central 1	70.69 ± 4.91	74.94 ± 3.90	0.001
Nasal 3	65.77 ± 2.62	68.72 ± 6.93	0.05
Nasal 6	63.96 ± 1.73	62.78 ± 11.33	0.6
Superior 3	64.31 ± 1.89	65.97 ± 2.22	0.003
Superior 6	64.50 ± 2.10	65.91 ± 2.30	0.02
Temporal 3	65.35 ± 2.04	66.94 ± 1.81	0.002
Temporal 6	63.65 ± 1.54	64.69 ± 2.08	0.04
Inferior 3	64.42 ± 2.32	65.25 ± 2.02	0.15
Inferior 6	63.58 ± 1.83	63.88 ± 2.25	0.58
Sum of all areas within 3	259.85 ± 7.43	259.34 ± 35.98	0.94
Sum of all areas within 6	255.69 ± 5.46	252.97 ± 35.24	0.70

**Table 4:** Comparison of mfERG amplitudes (nV/degree^2^) and amplitude ratios in both groups.

Grouped Responses	Group One	Group Two	*p* Value
R1	43.38 ± 12.04	41.18 ± 15.81	0.55
R2	24.66 ± 6.41	24.32 ± 7.80	0.86
R3	14.40 ± 3.77	14.206 ± 4.41	0.859
R4	10.25 ± 2.46	10.01 ± 2.78	0.73
R5	8.45 ± 2.09	8.37 ± 2.55	0.90
Ratios			
R1/R5	5.25 ± 1.42	5.00 ± 1.33	0.48
R2/R5	2.97 ± 0.65	2.99 ± 0.61	0.93
R3/R5	1.72 ± 0.31	1.74 ± 0.33	0.79
R4/R5	1.22 ± 0.12	1.22 ± 0.13	0.90

## Data Availability

Full access to the data is possible from the corresponding author.

## References

[1] Tan E.M., Cohen A.S., Fries J.F., Masi A.T., Mcshane D.J., Rothfield N.F., Schaller J.G., Talal N., Winchester R.J. (1982). The 1982 revised criteria for the classification of systemic lupus erythematosus. Arthritis Rheum..

[2] Stafford-Brady F.J., Urowitz M.B., Gladman D.D., Easterbrook M. (1988). Lupus retinopathy. Patterns, associations, and prognosis. Arthritis Rheum..

[3] Invernizzi A., Dell’Arti L., Leone G., Galimberti D., Garoli E., Moroni G., Santaniello A., Agarwal A., Viola F. (2017). Drusen-like Deposits in Young Adults Diagnosed with Systemic Lupus Erythematosus. Am. J. Ophthalmol..

[4] Baglio V., Gharbiya M., Balacco-Gabrieli C., Mascaro T., Gangemi C., Di Franco M., Pistolesi V., Morabito S., Pecci G., Pierucci A. (2011). Choroidopathy in patients with systemic lupus erythematosus with or without nephropathy. J. Nephrol..

[5] Sen S. (2021). Drusen-like deposits in systemic disorders: A point of convergence for nephrologists and ophthalmologists. J. Postgrad. Med..

[6] Basta F., Fasola F., Triantafyllias K., Schwarting A. (2020). Systemic Lupus Erythematosus (SLE) Therapy: The Old and the New. Rheumatol. Ther..

[7] Ding H.J., Denniston A.K., Rao V.K., Gordon C. (2016). Hydroxychloroquine-related retinal toxicity. Rheumatology.

[8] Marmor M.F., Kellner U., Lai T.Y., Melles R.B., Mieler W.F. (2016). Recommendations on Screening for Chloroquine and Hydroxychloroquine Retinopathy (2016 Revision). Ophthalmology.

[9] Gerth C., Hauser D., Delahunt P.B., Morse L.S., Werner J.S. (2003). Assessment of multifocal electroretinogram abnormalities and their relation to morphologic characteristics in patients with large drusen. Arch. Ophthalmol..

[10] Early Treatment Diabetic Retinopathy Study Research Group (1991). Grading diabetic retinopathy from stereoscopic color fundus photographs—An extension of the modified Airlie House classification. ETDRS report number 10. Ophthalmology.

[11] Invernizzi A., Pellegrini M., Acquistapace A., Benatti E., Erba S., Cozzi M., Cigada M., Viola F., Gillies M., Staurenghi G. (2018). Normative Data for Retinal-Layer Thickness Maps Generated by Spectral-Domain OCT in a White Population. Ophthalmol. Retin..

[12] Hood D.C., Bach M., Brigell M., Keating D., Kondo M., Lyons J.S., Marmor M.F., McCulloch D.L., Plamowski-Wolfe A.M. (2012). ISCEV standard for clinical multifocal electroretinography (mfERG) (2011 edition). Doc. Ophthalmol..

[13] Hanson J.V.M., Hediger M., Manogaran P., Landau K., Hagenbuch N., Schippling S., Gerth-Kahlert C. (2018). Outer Retinal Dysfunction in the Absence of Structural Abnormalities in Multiple Sclerosis. Investig. Ophthalmol. Vis. Sci..

[14] Robson A.G., Frishman L.J., Grigg J., Hamilton R., Jeffrey B.G., Kondo M., Li S., McCulloch D.L. (2022). ISCEV Standard for full-field clinical electroretinography (2022 update). Doc. Ophthalmol..

[15] Tsang A.C., Ahmadi S., Hamilton J., Gao J., Virgili G., Coupland S.G., Gottlieb C.C. (2019). The Diagnostic Utility of Multifocal Electroretinography in Detecting Chloroquine and Hydroxychloroquine Retinal Toxicity. Am. J. Ophthalmol..

[16] Dias-Santos A., Ferreira J.T., Pinheiro S., Cunha J.P., Alves M., Papoila A.L., Moraes-Fontes M.F., Proença R. (2020). Neurodegeneration in systemic lupus erythematosus: Layer by layer retinal study using optical coherence tomography. Int. J. Retin. Vitr..

[17] Işık M.U., Akmaz B., Akay F., Güven Y.Z., Solmaz D., Gercik Ö., Kabadayı G., Kurut I., Akar S. (2021). Evaluation of subclinical retinopathy and angiopathy with OCT and OCTA in patients with systemic lupus erythematosus. Int. Ophthalmol..

[18] Liu G.Y., Utset T.O., Bernard J.T. (2015). Retinal nerve fiber layer and macular thinning in systemic lupus erythematosus: An optical coherence tomography study comparing SLE and neuropsychiatric SLE. Lupus.

[19] Jones B.W., Pfeiffer R.L., Ferrell W.D., Watt C.B., Marmor M., Marc R.E. (2016). Retinal remodeling in human retinitis pigmentosa. Exp. Eye Res..

[20] Altinkaynak H., Duru N., Uysal B.S., Erten Ş., Kürkcüoğlu P.Z., Yüksel N., Duru Z., Çağıl N. (2016). Choroidal Thickness in Patients with Systemic Lupus Erythematosus Analyzed by Spectral-domain Optical Coherence Tomography. Ocul. Immunol. Inflamm..

[21] Dias-Santos A., Ferreira J.T., Pinheiro S., Cunha J.P., Alves M., Papoila A.L., Moraes-Fontes M.F., Proença R. (2019). Choroidal thickness changes in systemic lupus erythematosus patients. Clin. Ophthalmol..

[22] Kukan M., Driban M., Vupparaboina K.K., Schwarz S., Kitay A.M., Rasheed M.A., Busch C., Barthelmes D., Chhablani J., Al-Sheikh M. (2022). Structural Features of Patients with Drusen-like Deposits and Systemic Lupus Erythematosus. J. Clin. Med..

[23] Khan K.N., Mahroo O.A., Khan R.S., Mohamed M.D., McKibbin M., Bird A., Michaelides M., Tufail A., Moore A.T. (2016). Differentiating drusen: Drusen and drusen-like appearances associated with ageing, age-related macular degeneration, inherited eye disease and other pathological processes. Prog. Retin. Eye Res..

[24] Cheung L.K., Eaton A. (2013). Age-related macular degeneration. Pharmacotherapy.

[25] Pollreisz A., Reiter G.S., Bogunovic H., Baumann L., Jakob A., Schlanitz F.G., Sacu S., Owsley C., Sloan K.R., Curcio C.A. (2021). Topographic Distribution and Progression of Soft Drusen Volume in Age-Related Macular Degeneration Implicate Neurobiology of Fovea. Investig. Ophthalmol. Vis. Sci..

[26] Fortuna G., Brennan M.T. (2013). Systemic lupus erythematosus: Epidemiology, pathophysiology, manifestations, and management. Dent. Clin. N. Am..

[27] Ishizaki J., Saito K., Nawata M., Mizuno Y., Tokunaga M., Sawamukai N., Tamura M., Hirata S., Yamaoka K., Hasegawa H. (2015). Low complements and high titre of anti-Sm antibody as predictors of histopathologically proven silent lupus nephritis without abnormal urinalysis in patients with systemic lupus erythematosus. Rheumatology.

[28] Hoffmann M.B., Bach M., Kondo M., Li S., Walker S., Holopigian K., Viswanathan S., Robson A.G. (2021). ISCEV standard for clinical multifocal electroretinography (mfERG) (2021 update). Doc. Ophthalmol..

[29] Gerth C., Delahunt P.B., Alam S., Morse L.S., Werner J.S. (2006). Cone-mediated multifocal electroretinogram in age-related macular degeneration: Progression over a long-term follow-up. Arch. Ophthalmol..

[30] Gerth C. (2009). The role of the ERG in the diagnosis and treatment of Age-Related Macular Degeneration. Doc. Ophthalmol..

